# Controlling band alignments by artificial interface dipoles at perovskite heterointerfaces

**DOI:** 10.1038/ncomms7759

**Published:** 2015-04-07

**Authors:** Takeaki Yajima, Yasuyuki Hikita, Makoto Minohara, Christopher Bell, Julia A. Mundy, Lena F. Kourkoutis, David A. Muller, Hiroshi Kumigashira, Masaharu Oshima, Harold Y. Hwang

**Affiliations:** 1 Stanford Institute for Materials and Energy Sciences, SLAC National Accelerator Laboratory, Menlo Park, California 94025, USA; 2 Department of Materials Engineering, The University of Tokyo, Bunkyo, Tokyo 113-8656, Japan; 3 Geballe Laboratory for Advanced Materials, Department of Applied Physics, Stanford University, Stanford, California 94305, USA; 4 School of Applied and Engineering Physics, Cornell University, Ithaca, New York 14853, USA; 5 Kavli Institute at Cornell for Nanoscale Science, Ithaca, New York 14853, USA; 6 Photon Factory, High Energy Accelerator Research Organization, Tsukuba, Ibaraki 305-0801, Japan; 7 Department of Applied Chemistry, The University of Tokyo, Bunkyo, Tokyo 113-8656, Japan

## Abstract

The concept ‘the interface is the device' is embodied in a wide variety of interfacial electronic phenomena and associated applications in oxide materials, ranging from catalysts and clean energy systems to emerging multifunctional devices. Many device properties are defined by the band alignment, which is often influenced by interface dipoles. On the other hand, the ability to purposefully create and control interface dipoles is a relatively unexplored degree of freedom for perovskite oxides, which should be particularly effective for such ionic materials. Here we demonstrate tuning the band alignment in perovskite metal-semiconductor heterojunctions over a broad range of 1.7 eV. This is achieved by the insertion of positive or negative charges at the interface, and the resultant dipole formed by the induced screening charge. This approach can be broadly used in applications where decoupling the band alignment from the constituent work functions and electron affinities can enhance device functionality.

## Abstract (web summary)

Controlling the alignment of bands at oxide interfaces is crucial for developing them into useful devices. By inserting charges into the interface to generate dipoles, Yajima *et al.* show tuning of the band alignment between SrRuO_3_/Nb:SrTiO_3_ by 1.7 eV.

## Graphical abstract

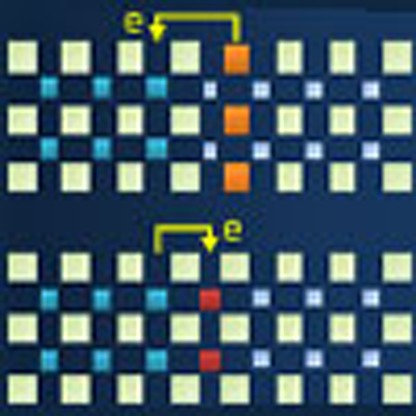

The rich physical properties of oxide interfaces present a formidable challenge, both in terms of scientific opportunities and for device design^1^. Several wide-gap oxide semiconductors, for example, are candidates for the next generation of optoelectronic devices^2^, taking advantage of their transparent nature^3^. More fundamentally, the discoveries of new electronic phases at oxide heterointerfaces highlight their strongly correlated electronic structure, intertwining charge, spin and orbital degrees of freedom^4^. Furthermore, oxide interfaces are central constituents in clean energy research, such as in photocatalysis^5^,^6^ and solar cells^7^. In all of these fields, a common principle which defines the device properties is the band alignment. In the simplest approximation, the band alignment between two materials is determined by the difference in their bulk electron affinities^8^. In reality, forming the interface often triggers the emergence of localized states (for example, metal-induced gap states), which dominate the band alignment over the electron affinity rule^9^. Oxides are interesting in this regard for a number of reasons. First, for ionic materials, the formation of in-gap (localized) interface states is far less likely, in contrast to the dangling bonds often found at covalent systems^10^,^11^. In addition, the recent development of atomic-scale perovskite heterostructures suggests the possibility to design dipole-induced modulations of the potential profile, which are expected to be larger for ions than for bond-centered covalent charge distributions.

In this work, we demonstrate this design possibility using SrRuO_3_/Nb:SrTiO_3_ perovskite metal/semiconductor Schottky heterojunctions; they are an ideal starting point in that the experimentally measured Schottky barrier height (SBH) is accurately given by the energy difference between the SrRuO_3_ metal work function (*Φ*) and the Nb:SrTiO_3_ n-type semiconductor electron affinity (*χ*)^12^,^13^. Our aim is to insert an artificial interface dipole, by which an additional modulation (Δ) of the potential can be achieved, leading to SBH=*Φ*−*χ*+Δ. By using the artificial interface dipole, we control the SBH at the SrRuO_3_/Nb:SrTiO_3_ Schottky junction over a broad range of 1.7 eV. This level of control, representing a change of ∼50% of the oxide semiconductor band gap, provides a compelling toolbox for oxide materials in a wide range of applications.

## Results

### Design of interface dipoles

**Figure 1:**
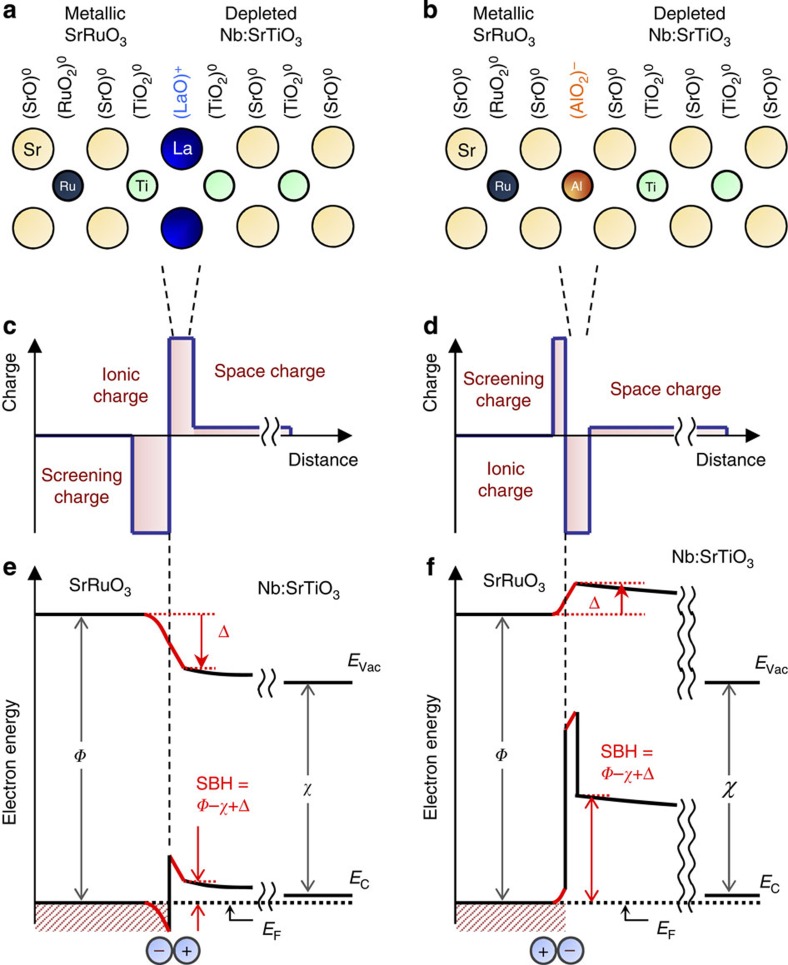
Interface dipole engineering at perovskite Schottky junctions. (**a**,**b**) Schematics of ionic alignments at SrRuO_3_/Nb:SrTiO_3_ heterointerfaces with (LaO)^+^ and (AlO_2_)^−^ layer insertion. As a function of distance from each heterointerface, (**c**,**d**) the charge distributions and (**e**,**f**) the electronic band diagrams are shown. The interface dipole between the ionic charge and the induced screening charge (**e**) decreases or (**f**) increases the SBH, both maintaining the relationship: SBH=*Φ*−*χ*+Δ. *E*_Vac_, *E*_C_ and *E*_F_ denote the vacuum level, the conduction band edge in Nb:SrTiO_3_ and the Fermi level of the whole system.

In the case of Schottky junctions, the artificial interface dipole can be formed in an elegant way: a monolayer of perovskite was inserted, which ionizes to give up an electron/hole which lies close to the layer as screening charge. Thus the dipole is formed by the ionized layer and the transferred electron/hole. For the ionized layers, (LaO)^+^ (Fig. 1a) and (AlO_2_)^−^ (Fig. 1b) are created by depositing just a few unit cells (u.c.) of either LaTiO_3_ or SrAlO_*x*_ between the SrRuO_3_ and Nb:SrTiO_3_ (see Methods section). In addition to the original space charge in the Nb:SrTiO_3_ and screening charge in the SrRuO_3_, additional ionic charge in the semiconductor and screening charges in the metal are introduced (Fig. 1c,d), forming a large atomic-scale electric dipole at the interface (Fig. 1e,f). This dipole modifies the band diagram according to its direction, and the resulting SBH (and Δ) can easily be detected by a host of well-established techniques.

### Electrical characterizations of SBHs

**Figure 2:**
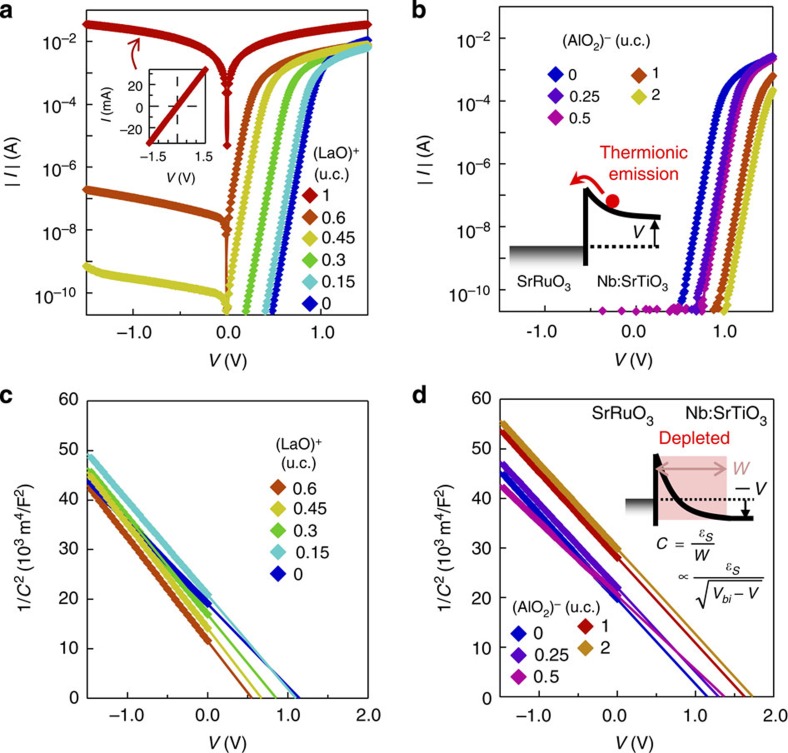
Electrical measurements of the SBH. (**a**,**b**) log|*I*|–*V* plots for SrRuO_3_/Nb:SrTiO_3_ Schottky junctions (0.091 mm^2^) with inserted (LaO)^+^ and (AlO_2_)^−^ layers, respectively. Inset in **a** shows the linear plot for the junction with 1 u.c. (LaO)^+^ layer insertion. (**c**,**d**) 1/*C*^2^–*V* plots for SrRuO_3_/Nb:SrTiO_3_ Schottky junctions with inserted (LaO)^+^ and (AlO_2_)^−^ layers. The amount of (LaO)^+^ and (AlO_2_)^−^ was varied from 0 u.c. to 1 u.c. (**a**,**c**) and from 0 u.c. to 2 u.c. (**b**,**d**), respectively. Solid lines in **c** and **d** are the linear extrapolations. Insets in **b** and **d** show schematic band diagrams for each measurement. For the latter, *ɛ*_S_, *W* and *V*_bi_ denote the semiconductor permittivity, the depletion width and the built-in potential.

Figure 2a,b show the current–voltage (*I*–*V*) characteristics for SrRuO_3_/Nb:SrTiO_3_ Schottky junctions with various (LaO)^+^ and (AlO_2_)^−^ layer coverage. The increase in forward bias current by (LaO)^+^ layer insertion (Fig. 2a) indicates the reduction of the SBH. A complete monolayer of (LaO)^+^ triggered a total collapse of the SBH, creating an Ohmic contact (Fig. 2a inset). In the case of (AlO_2_)^−^ layer insertion, the SBH is enhanced, as inferred from the suppression of the current for a given voltage as the ionic charge layer increases (Fig. 2b). For all samples, log |*I*| versus *V* is linear under forward bias, indicating transport via thermionic emission^14^ (Fig. 2b inset). From the intercept of extrapolating the forward bias region, the deduced SBH decreased from 1.4 to 0.7 eV after the insertion of 0.6 u.c. (LaO)^+^ layer, whereas with 2 u.c. of (AlO_2_)^−^, it increased to 1.9 eV. Here, it should be noted that above 0.6 u.c. (LaO)^+^ layer, the SBH is too low to be evaluated by electrical measurements. We also note that all junctions, even in cases of fractional layer insertion, showed ideal rectification with ideality factors near unity. This is reasonable since from atomic force microscopy, the characteristic lateral scale of the inhomogeneity in the fractional layers (<10 nm), is far smaller than the depletion width of the junctions (∼100 nm), such that the barrier heights are smoothed out microscopically and maintain a uniform electrostatic potential profile across the heterointerface^15^,^16^.

These SBH changes were confirmed by capacitance–voltage (*C–V*) measurements, as shown in Fig. 2c,d. All the junctions showed the characteristic linear relationship^14^ between 1/*C*^2^ and *V* (Fig. 2d inset). In this case, the abscissa intercept of the linear extrapolation corresponds to the built-in potential of these junctions, which is ∼0.1 eV smaller than the SBH at this doping density. Again we found a large decrease in the SBH (from 1.3 to 0.7 eV) with 0.6 u.c. (LaO)^+^ layer insertion, and a correspondingly large increase (to 1.8 eV) with 2 u.c. of (AlO_2_)^−^ layer insertion. Above 0.6 u.c. of (LaO)^+^ layer, the *C*–*V* data were no longer reliable because of the low impedance of the junctions.

### Spectroscopic characterizations of SBHs

**Figure 3:**
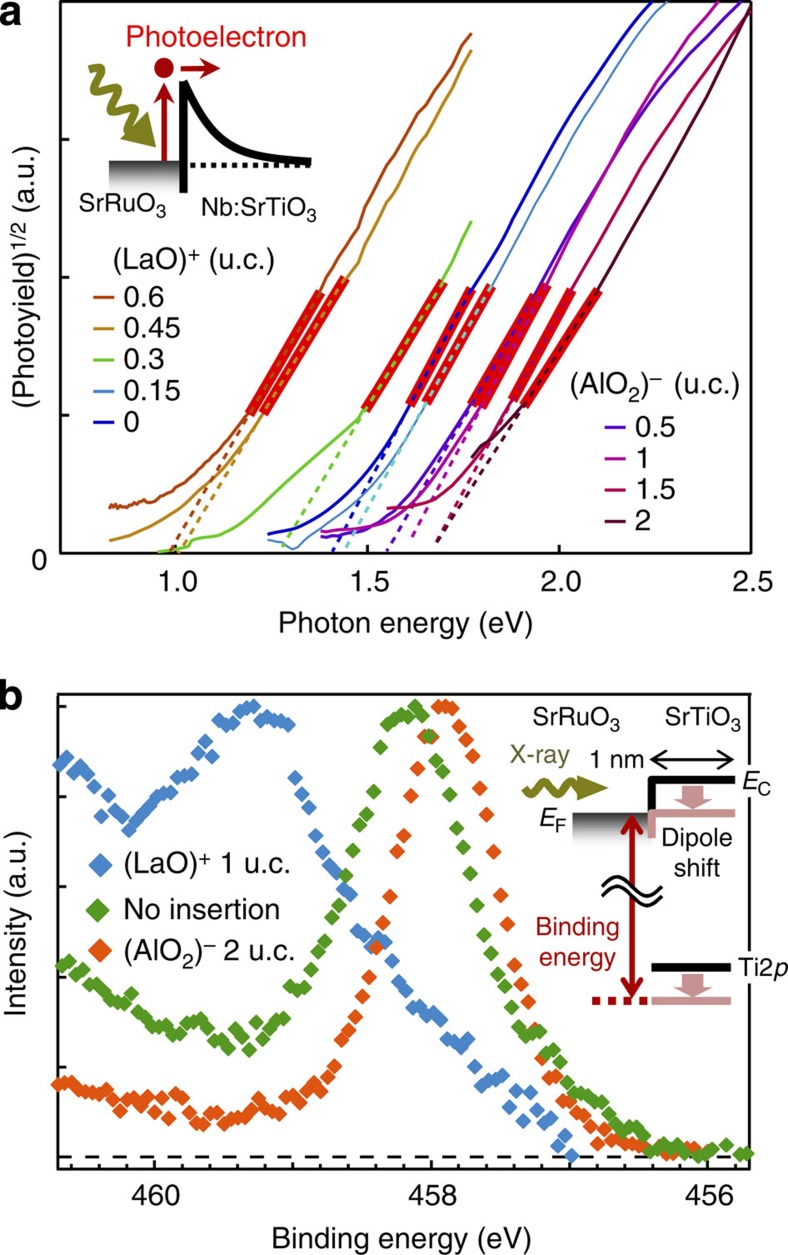
Photoelectric measurements of the SBH. (**a**,**b**) IPE and PES spectra for SrRuO_3_/Nb:SrTiO_3_ Schottky junctions with (LaO)^+^ and (AlO_2_)^−^ insertions. Red lines in **a** show the region over which the linear extrapolation (dashed lines) was carried out. Insets show the schematics of each measurement. The black arrow in **b** represents the detection width (∼1 nm).

**Figure 4:**
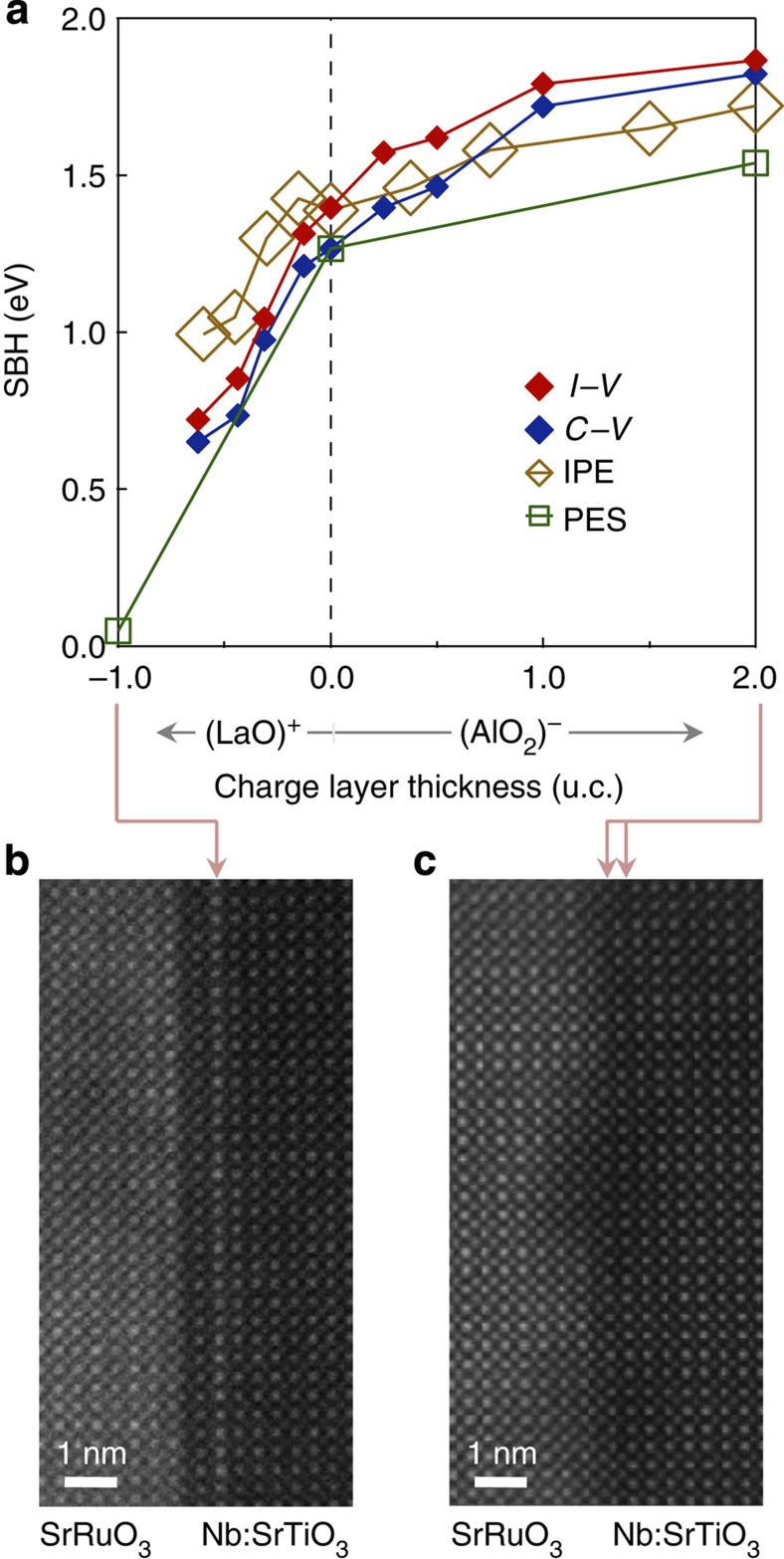
Summary of the SBH tuning. (**a**) SBH values obtained from *I*–*V*, *C*–*V*, IPE and PES measurements plotted as a function of interlayers, (LaO)^+^ and (AlO_2_)^−^. (**b**,**c**) High-angle annular dark-field-STEM images of SrRuO_3_/Nb:SrTiO_3_ heterointerfaces with (**b**) 1 u.c. (LaO)^+^ and (**c**) 2 u.c. (AlO_2_)^−^ insertions.

In addition to the electrical measurements, direct zero bias SBH measurements were performed using internal photoemission spectroscopy^14^ (IPE) and X-ray photoemission spectroscopy^12^ (PES). In IPE, photoexcited electrons on the metallic side traverse the interface and are detected as a photocurrent (Fig. 3a inset). The SBH corresponds to the extrapolated onset energy of the square root of the photoyield, (the photocurrent per incident photon)^17^. IPE spectra for all the SrRuO_3_/Nb:SrTiO_3_ Schottky junctions with (LaO)^+^ and (AlO_2_)^−^ layer insertions are plotted in Fig. 3a. From these data, we find that the SBH decreased from 1.4 to 1.0 eV for a (LaO)^+^ layer of 0.6 u.c., and increased to 1.7 eV with 2 u.c. of (AlO_2_)^−^ layer, in quantified agreement with the electrical measurements. Furthermore, three representative samples were measured with PES, using the Ti 2*p*_3/2_ core level shift with respect to the Fermi level *E*_F_ (Fig. 3b inset), providing a direct measure of the built-in potential^12^,^18^. As shown in Fig. 3b, it is qualitatively clear that the binding energy of the Ti 2*p*_3/2_ core level shifts to higher/lower energies when (LaO)^+^/(AlO_2_)^−^ layer is inserted, consistent with a decrease/increase in the SBH. More quantitatively, the PES spectra were decomposed into Ru 3*p*_3/2_ and Ti 2*p*_3/2_ components. The SBH shift was then obtained from the difference in the peak energies, after normalization to the 1.3 eV SBH value of the reference SrRuO_3_/Nb:SrTiO_3_ junction. These data, along with the values obtained from *I*–*V*, *C*–*V* and IPE, are plotted as a function of the interlayer in Fig. 4a. All the four methods show good quantified agreement, experimentally demonstrating proposed SBH tuning.

### Direct observations of the interfacial atomic structures

Of course, the real systems are not perfectly ideal; cross-section scanning transmission electron microscopy (STEM) images are given for the end-member interfaces (Fig. 4b,c). While La ions showed an atomically flat interlayer in Fig. 4b, Al ions sometimes formed patches as shown in Fig. 4c. Details of the growth of (LaO)^+^ and (AlO_2_)^−^ insertion layers are given elsewhere^19^,^20^. The aspect most relevant here is that the SrAlO_*x*_ used for (AlO_2_)^−^ insertion is not a stable perovskite in bulk, and can only be stabilized in thin layers using epitaxial strain^20^. It is likely that the intended (AlO_2_)^−^ layer is not completely oxidized even after post-annealing (see Methods section), possibly reflecting the recently discussed electrostatic stabilization of charged defects at oxide interfaces or surfaces^21^, resulting in the overall asymmetry of Fig. 4a and the apparent saturation of Schottky barrier shifts above a single-layer thickness.

## Discussion

The dipole formation by (LaO)^+^ insertion layer is reminiscent of the formation of two-dimensional electron gas by (LaO)^+^ insertion in the SrTiO_3_ matrix^19^,^22^. In this case, the replacement of La with Sm or Y may localize the electrons by forming a split-off lower Hubbard band a few hundred meV below the conduction band edge. However, in a Schottky junction, these localized electrons can be depleted in the Schottky barrier with the SBH of the order of 1 eV. Therefore, we predict that many other rare earth oxide layers including (SmO)^+^ and (YO)^+^ may also decrease the SBH in the same way as the (LaO)^+^ layer.

The total tunable range of ∼1.7 eV is remarkably large compared with other materials systems such as organics^23^,^24^ and conventional semiconductors^25^,^26^,^27^, where the magnitude of the interface dipole by self-assembled monolayers and highly-doped interlayers are limited by overlayer effects and small dopant solubility. The high efficiency of dipole engineering in oxide heterostructures demonstrated here originates from their ionic character as well as electronic and structural flexibility. Given such characteristics, oxide heterostructures represent an excellent platform where the band alignments are freely controlled by nano-scale interface engineering. This approach can be broadly applied to designing electrical contacts, interfaces optimized for charge separation and band lineups with respect to redox potentials, further expanding the functionality of this versatile class of materials.

## Methods

### Device fabrication

The Schottky junctions were fabricated by pulsed laser deposition using TiO_2_-terminated Nb:SrTiO_3_ (001) substrates (Nb=0.01 wt%). LaTiO_3_ (0–1 u.c.) was grown at an O_2_ pressure of 10^−5^ Torr, while SrAlO_*x*_ (0–2 u.c.) and SrRuO_3_ (40 u.c.) were grown at 0.3 Torr. The substrate temperature was 750 °C in all the cases. The thicknesses were controlled by using reflection high-energy electron diffraction oscillations and X-ray reflectivity. For Ohmic contacts, gold was evaporated onto the SrRuO_3_, and the whole structure was Ar-ion etched to form the junction area of 0.091 mm^2^. Post-annealing was performed in 760 Torr O_2_ at 300∼350 °C for 6 h to fill oxygen vacancies introduced by Ar-ion etching as well as originally existing in the SrAlO_*x*_. Although SrAlO_*x*_ is not a stable bulk perovskite, epitaxial stabilization is possible in an ultra-thin layer^20^, and by filling oxygen vacancies, we can ideally form negatively charged (AlO_2_)^−^ layer.

### SBH measurements

In the SBH estimate from *I*–*V* characteristics, the small deviation of the ideality factor (*n*) from unity was compensated by taking the intercept of the linear extrapolation between the measured SBH and *n* (ref. 16). The PES measurements using synchrotron radiation were carried out on beamline BL2C at the Photon Factory in KEK, Japan, with 800 eV incident photon energy. The PES spectra were recorded using a Scienta SES-2002 electron energy analyser with a total energy resolution of 150 meV. For the PES experiments, 2-nm-thick SrRuO_3_ films were used to minimize the attenuation of the emitted photoelectrons, and reduce the effect of the overlapping Ti 2*p*_2/3_ and Ru 3*p*_2/3_ core-level spectra. We confirmed that for this thinner SrRuO_3_, the electric properties were essentially unchanged.

### STEM imaging

STEM of cross-sectional samples was performed by high-angle annular dark-field imaging in an FEI Tecnai F20-ST.

## Author contributions

T.Y. performed the device fabrication, measurements and data analysis. Y.H., C.B. and H.Y.H. assisted with the planning, measurements and analysis of the study. M.M., H.K. and M.O. supported the PES measurements and analysis, and J.A.M., L.F.K. and D.A.M. performed the electron microscopy.

## Additional information

**How to cite this article:** Yajima, T. *et al.* Controlling band alignments by artificial interface dipoles at perovskite heterointerfaces. *Nat. Commun.* 6:6759 doi: 10.1038/ncomms7759 (2015).
